# Integration of transcriptomic and cytoarchitectonic data implicates a role for *MAOA* and *TAC1* in the limbic-cortical network

**DOI:** 10.1007/s00429-018-1620-6

**Published:** 2018-02-24

**Authors:** Sebastian Bludau, Thomas W. Mühleisen, Simon B. Eickhoff, Michael J. Hawrylycz, Sven Cichon, Katrin Amunts

**Affiliations:** 1 Research Centre Jülich, Institute of Neuroscience and Medicine (INM-1), 52425 Jülich, Germany; 2 Department of Biomedicine, University of Basel, 4031 Basel, Switzerland; 3 Research Centre Jülich, Institute of Neuroscience and Medicine (INM-7), 52425 Jülich, Germany; 4 Medical Faculty, Institute for Systems Neuroscience, Heinrich-Heine-University, 40225 Düsseldorf, Germany; 5 Allen Institute for Brain Science, Seattle, WA 98103 USA; 6 Institute of Medical Genetics and Pathology, University Hospital Basel, 4031 Basel, Switzerland; 7 Medical Faculty, C. and O. Vogt Institute for Brain Research, Heinrich-Heine-University, 40225 Düsseldorf, Germany; 8 JARA-Brain, Jülich Aachen Research Alliance, 52056 Aachen, Germany

**Keywords:** Differential gene expression, Cytoarchitecture, Brain maps, Multimodal analysis, Adult human brain

## Abstract

Decoding the chain from genes to cognition requires detailed insights how areas with specific gene activities and microanatomical architectures contribute to brain function and dysfunction. The Allen Human Brain Atlas contains regional gene expression data, while the JuBrain Atlas offers three-dimensional cytoarchitectonic maps reflecting interindividual variability. To date, an integrated framework that combines the analytical benefits of both scientific platforms towards a multi-level brain atlas of adult humans was not available. We have, therefore, developed JuGEx, a new method for integrating tissue transcriptome and cytoarchitectonic segregation. We investigated differential gene expression in two JuBrain areas of the frontal pole that we have structurally and functionally characterized in previous studies. Our results show a significant upregulation of *MAOA* and *TAC1* in the medial area frontopolaris which is a node in the limbic-cortical network and known to be susceptible for gray matter loss and behavioral dysfunction in patients with depression. The *MAOA* gene encodes an enzyme which is involved in the catabolism of dopamine, norepinephrine, serotonin, and other monoaminergic neurotransmitters. The *TAC1* locus generates hormones that play a role in neuron excitations and behavioral responses. Overall, JuGEx provides a new tool for the scientific community that empowers research from basic, cognitive and clinical neuroscience in brain regions and disease models with regard to gene expression.

**Electronic supplementary material:**

The online version of this article (10.1007/s00429-018-1620-6) contains supplementary material, which is available to authorized users.

## Introduction

The Allen Human Brain Atlas (AllenBrain) comprises tools and data resources for the daily work of neuroscientists who focus on genes and their expression patterns in tissues and cells of the adult human brain (Hawrylycz et al. 2015). The messenger RNA (mRNA) expression reflects the transcriptomic architecture of six postmortem brains (six left and two right hemispheres) labeled with anatomical landmarks according to gyral and sulcal patterns; the data derive from anisotropically distributed tissue samples (TSs) computationally registered to the coordinate space of the Montreal Neuroscience Institute 152 (MNI_152_) reference brain (Evans et al. 2012). The three-dimensional (3D) cytoarchitectonic maps of the Jülich–Düsseldorf Brain Atlas (JuBrain; Amunts and Zilles 2015) are based on microstructural mappings generated by an observer-independent approach in ten post-mortem brains (Schleicher et al. 1999); the maps display probability distributions as a measure of the interindividual variability of the anatomical areas in space and extent (Zilles and Amunts 2010; Amunts and Zilles 2015). To fit to AllenBrain data, we registered JuBrain data to the MNI_152_ space which is used as a common reference space. JuBrain maps have frequently been applied in structural and functional neuroimaging (sMRI, fMRI) studies to facilitate data analyses on a sound biological basis (Bludau et al. 2014, 2016; Lorenz et al. 2015; Henssen et al. 2016).

**Fig. 1:**
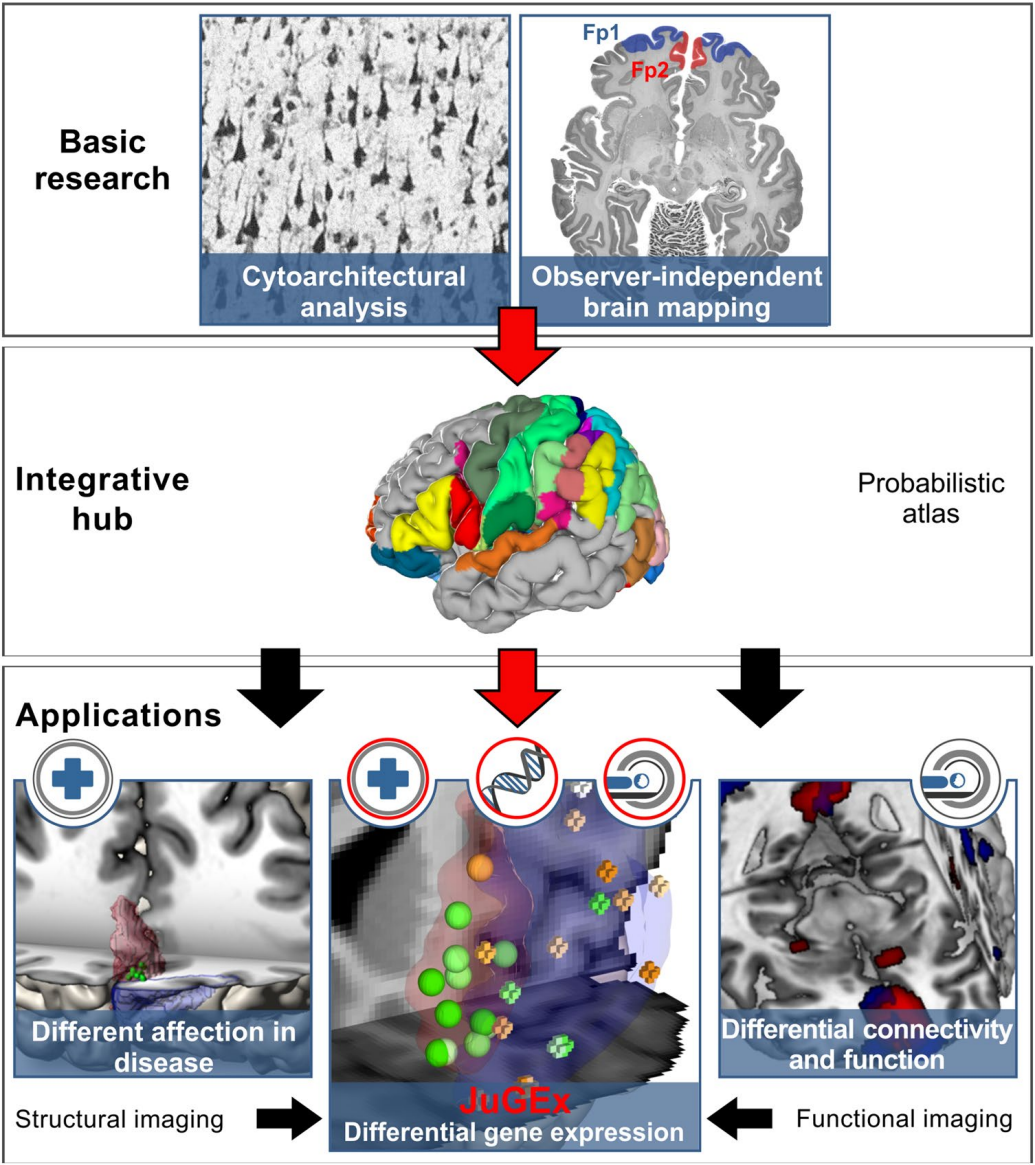
JuGEx links cytoarchitecture and gene expression to investigate multilevel human brain organization. Observer-independent mapping, which systematically quantifies regional patterns of densely packed cells, has facilitated to identify areas that are now part of the 3D atlas JuBrain (upper and middle row; Schleicher et al. 1999; Zilles and Amunts 2010; Amunts and Zilles 2015). Such probabilistic maps of structurally and functionally specialized tissues represent an integrating point to other aspects of brain architecture, e.g., connectivity, resting-state connectivity and brain activations (lower row). Each level of information provides new insights into brain organization and helps to analyze the different aspects of the areas under healthy and pathological conditions

**Fig. 2:**
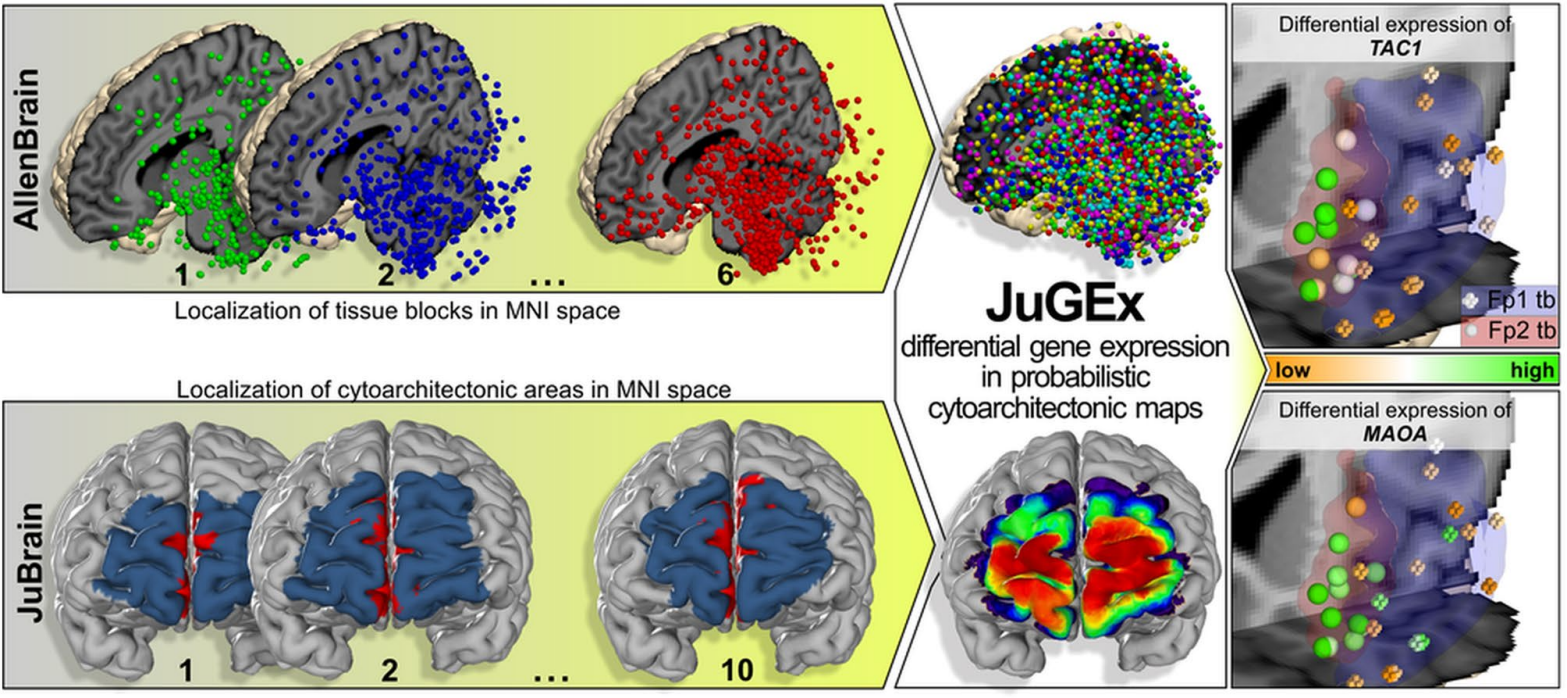
JuGEx is an integrated framework of the AllenBrain and JuBrain atlases for statistical analysis of differential gene expression in the adult human brain. The data of both atlases are based on postmortem brains that have been scanned by MRI and transformed to a common reference brain (MNI_152_). The upper row displays the positions of TSs (colored spheres) in three of six donor brains from AllenBrain. The transcriptional expression has been quantified in these TSs using oligoprobes of Agilent microarrays. The lower row shows an example of microstructural information quantified in the frontal pole areas Fp1 (blue) and Fp2 (red) in three of ten donor brains from the Jülich–Düsseldorf brain collection. To combine the data from the two modalities (transcriptom and cytoarchitecture), probabilistic JuBrain maps are used as masks to filter the TS-specific expression information as starting point for subsequent statistical analysis of differential expression of genes (JuGEx column). In the example of use (most right column), we investigated the expression patterns of 25 candidate genes for MDD in the lateral Fp1 (blue) and the medial Fp2 (red). The cut outs are located at the frontal pole and display the left hemispheric part of area Fp1 and area Fp2. Analyses were performed using a permuted *n*-way ANOVA. Results show that *TAC1* (upper panel, *p* = 0.0216) and *MAOA* (lower panel, *p* = 0.0292) are significantly stronger expressed in tissue samples of Fp2 (spheres) than in those of Fp1 (crosses). The level of mRNA expression is indicated by a gradient running from orange (lower expression) to green (higher expression)

Up to now, an integrated framework for combined analyses towards a multi-level human brain atlas (Amunts et al. 2014) has not been developed so far (Fig. 1). To achieve this, we have developed JuGEx (**Ju**Brain **G**ene **Ex**pression), a user-friendly workflow based on graphical user interfaces (GUIs) that enables multiple ways of analyses of differential gene expression and cytoarchitectonic data (Fig. 2). As an example of use, we analyzed expression patterns of candidate genes for major depressive disorder (MDD) in the brain’s frontal pole. The rationale behind this was that this region is structurally and functionally segregated and plays an important role in the pathophysiology of MDD as we have recently demonstrated (Bludau et al. 2014, 2016). Historically, the frontal pole has been associated with Brodmann’s area 10 (BA10; Brodmann 1909). However, we have identified that BA10’s cytoarchitecture is heterogeneous and consists of two distinct areas—a lateral area frontopolaris 1 (Fp1) and a medial area frontopolaris 2 (Fp2; Bludau et al. 2014). This segregation is supported by neuroimaging data showing different patterns of functional activation for the medial Fp2 (involved in socio-affective behavior) as compared to the lateral Fp1 (involved in cognition) in control subjects (Ramnani and Owen 2004; Burgess et al. 2007; Gilbert et al. 2010; Bludau et al. 2014). In this regard, Fp2 but not Fp1 can be considered as a node in the dysfunctional network model of MDD (Mayberg 2003). So far, it is has been unknown, whether this structural and functional heterogeneity of the frontal pole is reflected by region-specific differential expression of genes that contribute to susceptiblity of MDD.

The added value of JuGex is that different levels of information on brain architecture, e.g., structural and functional connectivity, brain activations, and neurotransmitter receptor density, can now be supplemented by transcriptional information to enlight biological aspects of brain organization and its diseases, spatially referring to the cytoarchitectonic JuBrain atlas. This allows analysis beyond approaches which rely on the traditional segregation of the brain into sulci and gyri, thereby lumping together functionally different microstructural areas.

## Materials and methods

### Registration of JuBrain and AllenBrain data

The JuBrain atlas is based on mappings of cytoarchitectonic areas in ten postmortem brains (five females, five males, 30–86 years) using an observer-independent technique (Schleicher et al. 1999). The JuBrain maps describe, for each voxel of a reference space, the probability with which a certain area can be found at this position (Zilles and Amunts 2010). Originally, JuBrain data have been anchored to the MNI-Colin27 reference space. For JuGex, we registered the maps to the MNI_152_ space which is used by AllenBrain. In fact, we computed a linear and non-linear registration between the Colin and ICBM 2009c non-linear asymmetric data and applied the computed transformations to the individual maps. For the computation, we used the software that had been used for the original registration of the postmortem brains of the JuBrain atlas to the Colin space (Hömke 2006). Data of the AllenBrain atlas were retrieved throuth the AllenBrain API. The registration of these data to MNI_152_ had been performed by AllenBrain developers using the Freesurfer recon pipeline and a manually initialized affine transformation in cases where the initial linear MRI-to-MNI space transformation estimation procedure failed (Hawrylycz et al. 2012). The affine transformation was estimated through the placement of homologous landmark pairs in register, as part of the MNI/MINC toolbox available at https://www.bic.mni.mcgill.ca/ServicesSoftware/HomePage.

### Workflow and graphical user interfaces of JuGex

**Fig. 3:**
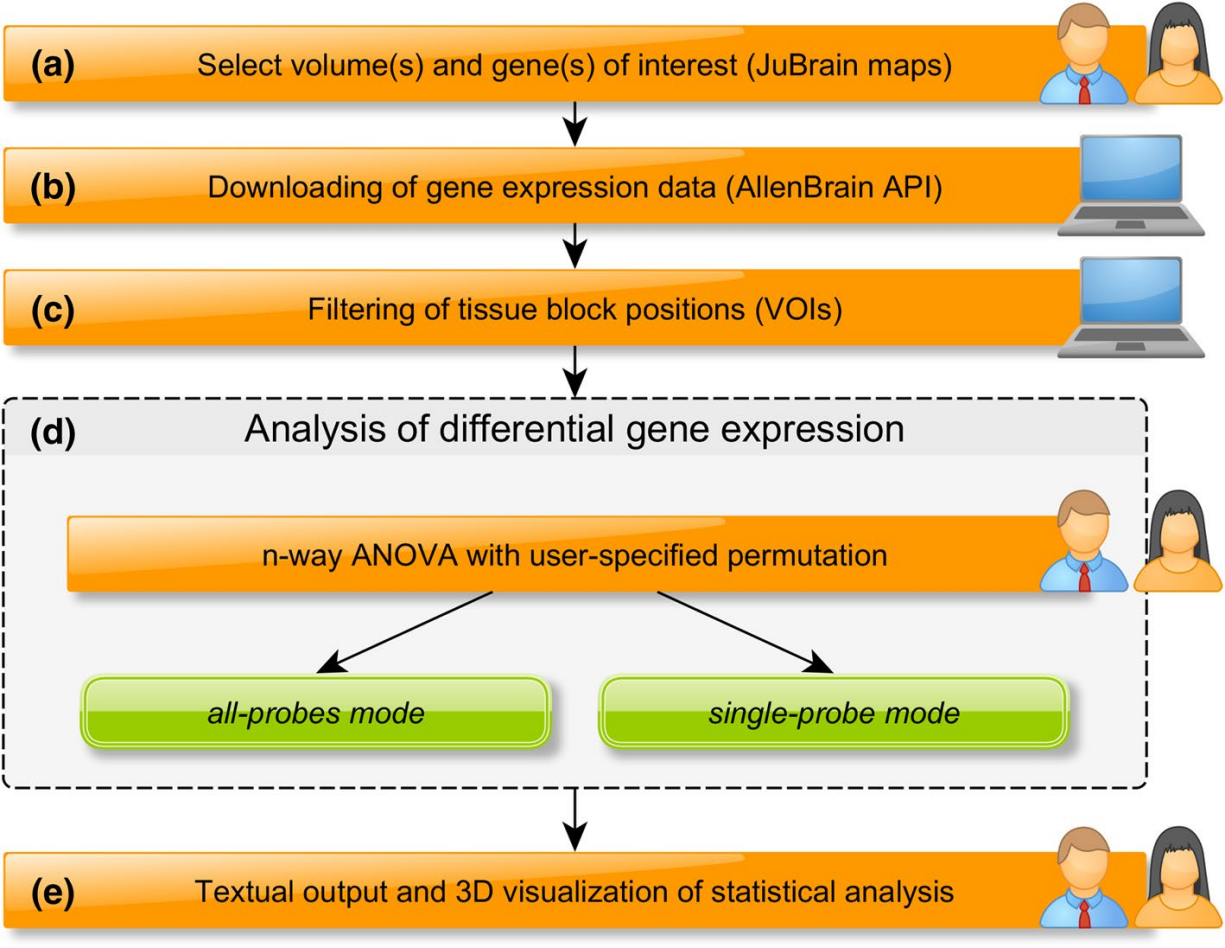
JuGEx workflow **a**–**c** to configure an experiment, the user enters the genes and selects the VOIs according to the Entrez gene nomenclature and JuBrain maps. Then, the corresponding mRNA expression levels (*z*-scores) are downloaded from AllenBrain (API) and TSs are filtered using the VOIs as masks. **d, e** In the statistical analysis, the user can choose between the all-probe and single-probe modes to analyze differential expression of individual genes in the VOIs. In addition to the textual output, the user can visualize the expression levels from either analysis in a 3D graphics for each gene

The workflow of JuGex comprises four main steps. First, using the GUI *Configuration*, the user selects the genes and two volumes of interest (VOIs), which should be compared with respect to expression differences (Fig. 3a). VOIs can be single JuBrain maps, a merge of JuBrain maps (neighboring or distant), AllenBrain labels (gyrally and sulcally defined), and VOIs from sMRI, respectively, fMRI findings (Fig. 1). The first version of JuGex only offers individual JuBrain maps and AllenBrain labels in the MNI_152_ space. To merge and register any other VOI to this reference space, we ask the user to use established imaging tools before uploading it to JuGex. Map thresholds that determine VOI sizes can be selected between 10–100%; the larger the threshold, the smaller the VOIs; default is set at 20%. Smaller VOIs reflect brain areas with a more rigorous approach and exclude portions with high interindividual variability.

Second, gene expression data are downloaded from the AllenBrain platform through an application programming interface (API; Fig. 3b). The full data comprise expression levels of 20,787 Entrez genes and their transcript isoforms measured by 58,692 mRNA oligoprobes of Agilent microarrays. The mRNA molecules derive from 3682 TSs of all available donors (one female, five males, 24–57 years of age), in particular, six left and two right brain hemispheres. To enable a direct comparison of expression patterns between VOIs in different brains, JuGex uses relative expression values that have been *z*-score normalized by AllenBrain developers (Hawrylycz et al. 2012, 2015).

Third, expression data are filtered using the VOIs as masks in each brain of AllenBrain, i.e., only those expression data are included in the subsequent analyses, which stem from TSs within the VOIs (Fig. 3c). This is possible since both JuBrain and AllenBrain data are located in the same reference space (MNI_152_). During the procedure, VOI-specific labels, e.g. VOI1 and VOI2, are assigned to TSs (TSLs) for the subsequent statistical procedure. Up to this point, the workflow has generated a condensed set of regional gene expression data.

Fourth, the user can choose between the two analytical modes using the GUI *Analysis*. The single-probe mode focuses on specific isoforms/splice variants by analyzing the expression level of each oligoprobe individually, with the benefit that the user can investigate strongly, moderately or weakly expressed gene variants (transcript isoforms) in the VOIs. This means that *z*-scores from TSs of VOI1 are compared against corresponding data from VOI2. Since the expression level of most genes have been measured by several different probes, the all-probes mode averages the probe data using a winsorized mean; this approach provides a robust estimation of the expression since *z*-score outliers can be excluded (Wilcox and Keselman 2003). We have set a lower threshold of 10% and an upper threshold of 90%, as proposed by a previous study of gene expression in human brain using microarrays (Ramasamy et al. 2014). In either mode, the *z*-scores are introduced to a series of *n*-way analysis of variance (ANOVA). The *z*-scores of the TSs are used as dependent variable and the TSLs, donors, age, sex, ethnicity are used as independent variables (factors); TSLs are the factor-of-interest (Fig. 3d).

The statistical procedure comprises a reference statistic, permuted statistics, and a correction for multiple comparisons (Box 1): the *F* value of the first *n*-way ANOVA uses the original TSLs and serves as a reference statistic. The subsequent, adapted *n*-way ANOVAs are run with a user-specified number of permutations (default 10,000 rounds) and randomly shuffle the TSLs between VOIs under the assumption of label exchangeability. This means that the permuted statistics are then compared against the reference statistic to differentiate between expression differences produced by chance (false-positive) and those which show a significant difference between the analyzed VOIs (true-positive). Finally, the nominal *p* values are adjusted for the number of multiple comparisons using a family-wise error (FWE) correction. This implies that JuGex corrects the results for the number of selected genes (all-probe mode) respectively the number of analyzed probes (single-probe mode) depending on the chosen analysis mode (Supplementary Fig. 1). That is, the maximum *F* value per permutation across all investigated genes (probes in single-probe mode) is extracted. Then, the corresponding *p* values are calculated relative to the distribution of the maximum *F* values across all replications. This results in a FWE correction over all analyzed genes (probes in single-probe mode). We consider a *p* value smaller than 0.05 as a significant difference between the *z*-scores of TSs in the compared VOIs. The statistical results can be visualized in the MNI_152_ space by the implemented GUI *Visualization* (Fig. 3e). An additional textual output contains data from the performed analysis: *z*-scores (probe-specific expression levels or winsorized mean expression levels) from each TS as well as all FWE- and nominal *p* values for individual follow-up analysis and plotting.


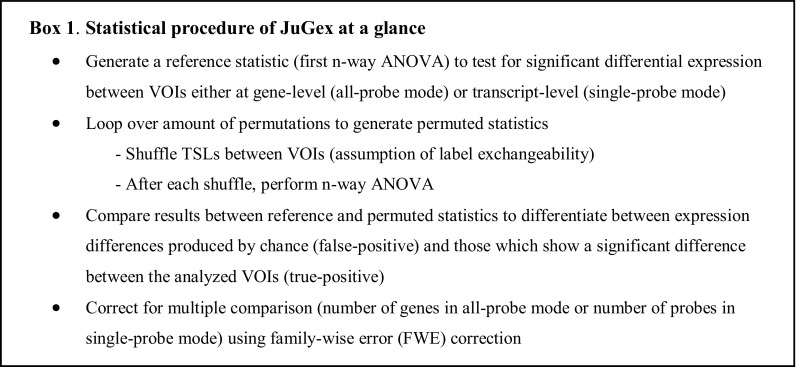


### Selection of genes

For the main analysis, candidate genes for MDD were drawn from the technical white paper “Complete List of Genes Characterized by in situ Hybridization in Adult Human Brain Studies” published by the Allen Brain Institute and available at https://help.brain-map.org/download/attachments/2818165/HBA_ISH_GeneList.pdf?version=1&modificationDate=1348783035873&api=v2. All genes from the category “disease” showing the flag “depression” were included; there were no exclusion criteria. The procedure yielded a number of 25 candidate genes for MDD.

To perform analyses for assessment of JuGex results, two independent sets of genes were assembled as negative controls (Supplementary Table 1b, c). The first set was randomly drawn from the AllenBrain genes and consisted of 25 genes (random genes). The second set was intentionally drawn from the AllenBrain genes and comprised 14 genes from genome-wide association studies (GWAS) of eye, hair, and skin coloration (color genes; EMBL-EBI GWAS catalog, as of 21st November 2017, available at https://www.ebi.ac.uk/gwas/).

### Code availability

The JuGEx workflow and the GUIs are based on a script distribution that was coded in Matlab (version R2015b, 64bit; The MathWorks). All codes are freely available through a download at http://www.fz-juelich.de/inm/inm-1/jugex. An overview about the currently available JuBrain maps for the whole brain is described at https://www.jubrain.fz-juelich.de.

## Results

In the main analysis, we analyzed the expression of 25 MDD genes in the frontal pole using all available brains from AllenBrain (Supplementary Table 1a); body donors were free of psychiatric or neurological diseases and intoxications (Hawrylycz et al. 2015). All steps of JuGEx were executed under default settings (Supplementary Table 2). The downloading and filtering steps extracted 12 TSs mapped to left Fp2 and 18 TSs mapped to left Fp1. The all-probes analysis identified two differentially expressed genes after the correction for multiple comparisons: the best result was achieved for *TAC1* (*p*_FWE_ = 0.0216) assessed by 13 oligoprobes, while the second best result was for *MAOA* (*p*_FWE_ = 0.0292) assessed by 31 oligoprobes (Supplementary Fig. 2a). The 3D visualization of the winsorized means across the oligoprobes revealed that both genes were significantly higher expressed in the medial Fp2 compared to the lateral Fp1. In the single-probe analysis, the most significant result was detected for CUST_2036_PI417557136 (*p*_FWE_ = 0.0475) that targets three protein-encoding transcript-isoforms of *TAC1* (ENST00000319273.9, ENST00000346867.4, ENST00000350485.8) (Supplementary Fig. 2b; Supplementary Table 3). *MAOA* did not show a significant result for a single isoform. However, 17 of the 31 probes showed nominal associations (*p*_nominal_ < 0.05, uncorrected; Supplementary Fig. 2c).

In subsequent analyses, we assessed the results of the main analysis. For this purpose, we separately investigated 25 random genes and 14 color genes using the same settings as for the main analysis. Neither of the two control analyses showed a significant differential expression in Fp1 compared to Fp2 (*p*_FWE_ > 0.05).

## Discussion

JuGex links the two atlas systems of AllenBrain and JuBrain. Here, we have demonstrated that JuGEx is a valuable novel tool for analysis of differential gene expression in conjunction with precise topographical information on a sound microstructural basis.

In the example of use, we identified two differentially expressed genes, each showing an upregulation in Fp2 compared to Fp1. *TAC1* (tachykinin precursor 1) maps to chromosome 7q21.3. The gene locus generates neurokinin 1 (alias substance P), neurokinin A, neuropeptide K, and neuropeptide gamma. Tachykinins are hormones that mediate a variety of physiological functions in the body by binding to their receptors. In the brain, the tachykinin system is involved in the excitation of neurons and induction of behavioral responses. *MAOA* (monoamine oxidase A) is located on chromosome Xp11.3. The enzyme plays a key role in the degradation of monoaminergic neurotransmitters such as dopamine, norepinephrine, and serotonin. In models of depression-related behavior, mice with a homozygous deletion of *tac1* (*tac1*^−/−^) were more active than wildtype mice, i.e., they behaved like wildtype mice under medication with an antidepressant, as reviewed by Bilkei-Gorzo and Zimmer (2005). Interestingly, *tac*^−/−^ mutants behave similiar to *maoa*^−/−^ mutants. Therefore, the tachykinin system is under consideration as promising drug target, while the pharmacological blockade of the catabolic *MAOA* enzyme has already been established for the therapy of depression (Bilkei-Gorzo and Zimmer 2005).

There are only a few studies that have investigated expression of *TAC1* in human brains (Guilloux et al. 2012; Malki et al. 2015). One study reported a significantly lower expression of *TAC1* in the frontal pole of MDD patients without separating this effect between its medial and lateral areas (Malki et al. 2015). One can therefore assume that the higher *TAC1* expression in controls was mainly driven by transcript molecules in the medial Fp2. This hypothesis may help to design molecular genetic assays for validation in independent samples of postmortem tissue in the wet lab, for instance, a quantitative polymerase chain reaction targeting *TAC1* transcripts. *MAOA* expression has not been investigated specifically in the frontal pole but its contribution to the MDD pathogenesis has been investigated in numerous publications (Meyer et al. 2009; Lung et al. 2011). Especially Fp2, in which *MAOA* expression was higher compared to Fp1, is discussed as being a genetically modulated regulatory area for emotional stimulation and social behavior (Buckholtz et al. 2008). Moreover, a length-polymorphism in the *MAOA* promoter is associated with the degree of functional connectivity between the amygdala and the ventral medial prefrontal cortex, a macroanatomical label that includes Fp2 (Buckholtz et al. 2008).

It is important to keep in mind that the gene expression data derive from tissue samples and reflect the region-specific composition of neuronal, glial, and endothelial cell types. Therefore, we cannot exclude that the reported expression differences between Fp1 and Fp2 are influenced by normal differences in cell-type proportions. This means that the results cannot provide evidence on a cellular level, but they can be taken as a starting point for follow-up investigations using wet lab methods. Together with the Fp2-specific gray matter atrophy in the left hemisphere of MDD patients (Bludau et al. 2016), the differential expression of *TAC1* and *MAOA* between Fp2 and Fp1 provides a further argument to include Fp2 as a node in the dysfunctional network of MDD.

To evaluate the methodological robustness and the biological specificity of our findings, we performed two control analyses and took a closer look at the probes of the MDD genes. Neither the genes of a random selection nor the genes of body coloration achieved a single significant result. Both negative results strongly suggest that knowledge about genes, MDD, and brain regions plays an important role for the outcome of our study. Additionally, the 13 probes of *TAC1* and the 31 probes of *MAOA* probes showed comparable expression levels indicating stable measurements which are not driven by outliers (Supplementary Fig. 2). Moreover, MDD genes with a smaller, the same, or a larger number of probes do not yield a significant result per se: *SST* encoding the peptide hormone somatostatin (4 probes), *HTR1D* encoding a serotonin receptor (13 probes), or *CNR1* encoding a cannabinoid receptor (89 probes). Of note, *CNR1* belongs to the genes with the largest numbers of probes among the AllenBrain genes. Overall, we conclude that our findings for *MAOA* and *TAC1* are not affected by systematic bias.

The strength of JuGex is an easy and rapid way of testing region-specific hypothesis and/or to generate new hypotheses for wet lab experiments. However, it cannot provide specific evidence on a cellular level since the investigated TSs consist of multiple cell types. Our workflow allows designing investigations either from a genetic perspective (candidate genes) or from an anatomical point of view (probabilistic cytoarchitectonic maps) in a common reference space. The application shows the capability of JuGEx to identify differentially expressed genes in structurally and functionally specialized tissues (areas Fp1/Fp2) as a prerequisite to understand the biological processes involved in brain function and dysfunction. Moreover, the cytoarchitectonic maps of JuBrain atlas in the format of the MNI_152_ reference brain represent a hub for integrating other aspects of cortical architecture such as connectivity, resting-state connectivity, task-dependent and activation maps (Fig. 1). To allow convergence of the manifold facets of brain organization in a common spatial framework, our tool paves the way to achieve a more comprehensive understanding of human brain function (Amunts and Zilles 2015). JuGex is publicly available to empower research from basic, cognitive and clinical neuroscience in further brain regions and disease models with regard to gene expression.

## Electronic supplementary material

Below is the link to the electronic supplementary material.

Supplementary Figure 1. Differential gene expression analysis. The left and right panels illustrate exemplary positions of TSs (TS_1_-TS_k_) within the user-configured VOIs. In the corresponding tables, the columns single-probe mode show the oligoprobe signals (OP_1_-OP_n_) across the filtered TSs. The columns all-probe mode display the subsequently calculated winsorized means of the oligoprobe signals for the selected genes (gene_1_-gene_n_). Finally, the resulting data of the VOIs, here Fp1 and Fp2 as examples, are introduced to the differential gene expression analysis using a *n*-way ANOVA approach (PNG 178 KB)

Supplementary Figure 2. Box- and whisker plots of differential gene expressions. (**a**) Expression levels (*z*-scores) from the all-probes mode for the 25 investigated candidate genes. *TAC1* and *MAOA* achieved the significant results. Red boxes: Fp2; blue boxes: Fp1; left y-axis: AllenBrain identifier; right y-axis: Entrez gene symbol. **(b)** Expression levels (*z*-scores) from the single-probe mode for *TAC1*, and **(c)** for *MAOA* (left y-axis: AllenBrain identifier; right y-axis: oligoprobe identifier) (PDF 1378 KB)

Supplementary Movie 1. Animated visualization showing the localization of the investigated TSs in the 3D reference space and their differential gene expressions (MP4 25817 KB)

Supplementary Table 1. Overview of all genes analyzed in the present study. (**a**) The 25 candidate genes for MDD including oligoprobe identifier (probe_id), gene (gene_symbol), and Entrez gene identifier (entrez_id), (**b**) the 25 random genes, (**c**) the 14 color genes and the original references from which they were selected (PDF 208 KB)

Supplementary Table 2. Description of the default settings used in the standard workflow. (**a**) GUI *Configuration*, (**b**) GUI *Analysis*, and (**c**) GUI *Visualization* (DOCX 136 KB)

Supplementary Table 3. Textual output of the result from the example of use generated by the Matlab console. The index indicates the line number from the input gene list (PDF 17 KB)
