# Supplementary figures and images for: Integration of transcriptomic and cytoarchitectonic data implicates a role for MAOA and TAC1 in the limbic-cortical network
Source: Brain Struct Funct. 2018 Feb 24;223(5):2335–42. doi: 10.1007/s00429-018-1620-6 (PMC5968065; doi:10.1007/s00429-018-1620-6)

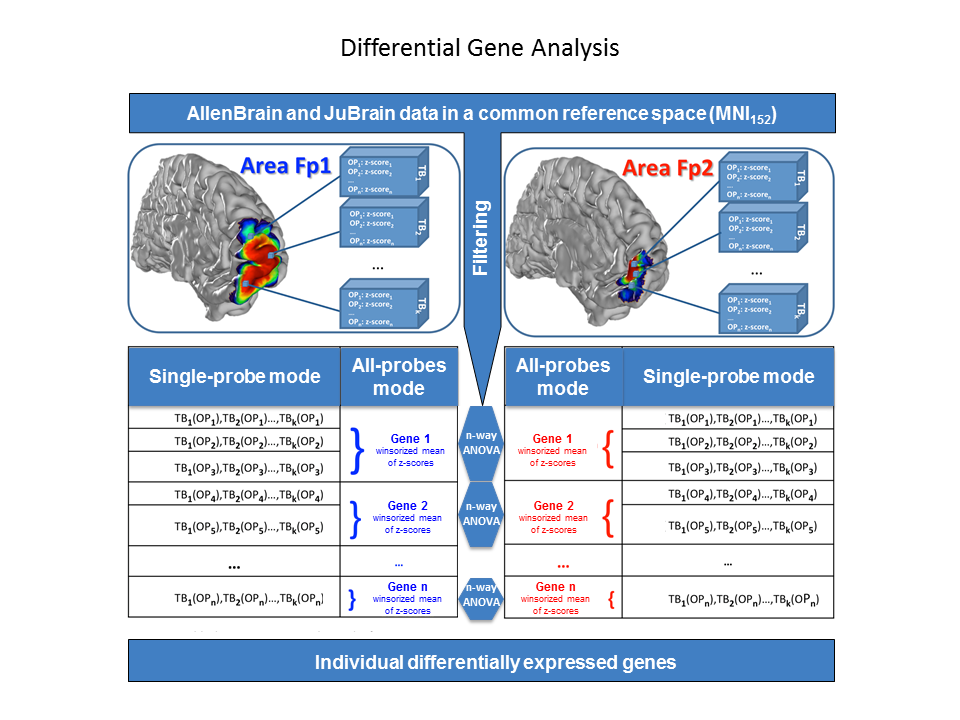

Supplement: Supplementary file 1 — Supplementary Figure 1. Differential gene expression analysis. The left and right panels illustrate exemplary positions of TSs (TS1-TSk) within the user-configured VOIs. In the corresponding tables, the columns single-probe mode show the oligoprobe signals (OP1-OPn) across the filtered TSs. The columns all-probe mode display the subsequently calculated winsorized means of the oligoprobe signals for the selected genes (gene1-genen). Finally, the resulting data of the VOIs, here Fp1 and Fp2 as examples, are introduced to the differential gene expression analysis using a n-way ANOVA approach (PNG 178 KB) [file 429_2018_1620_MOESM1_ESM.png]

a)

25 Genes (All-probes mode)

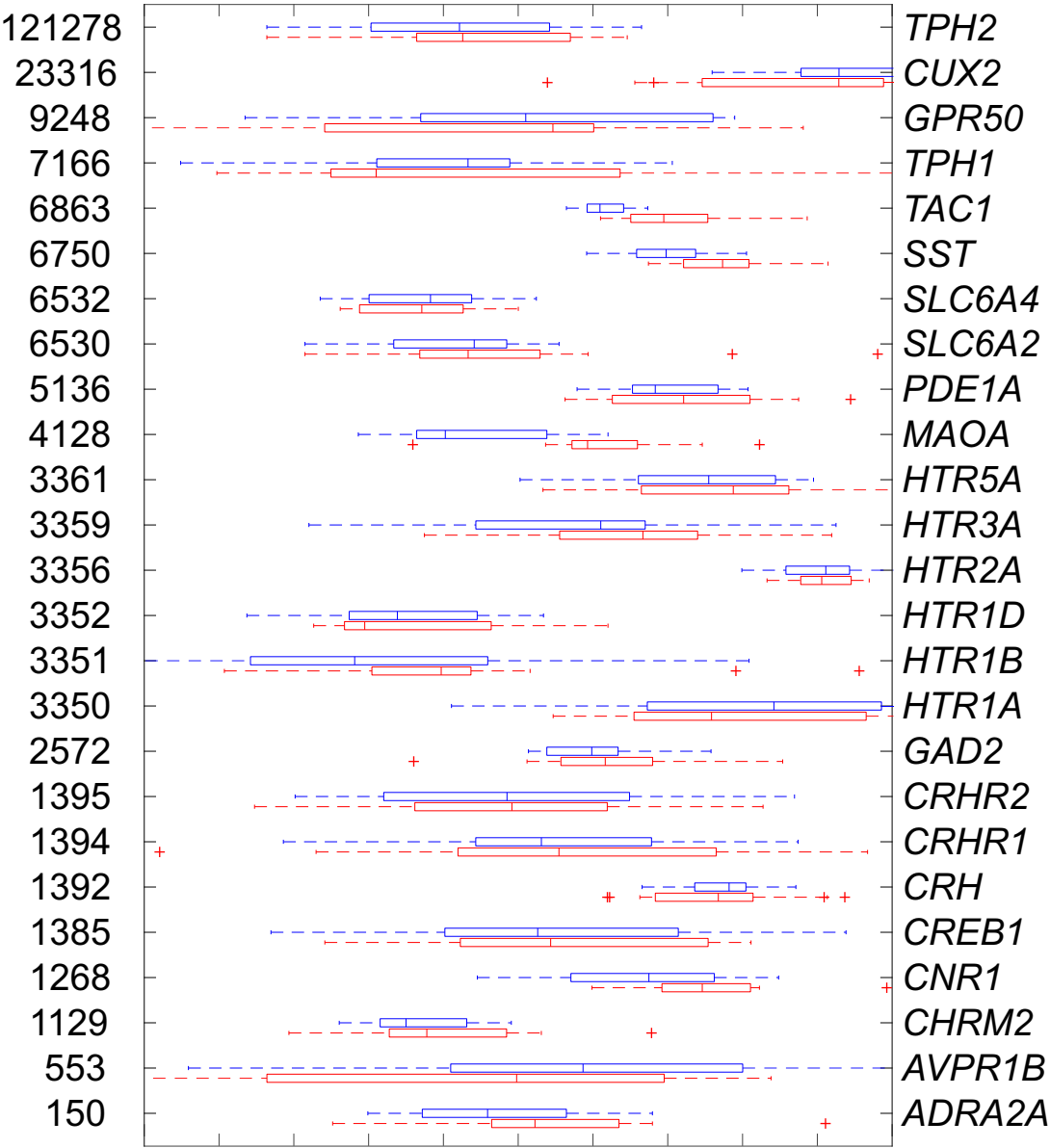

b)

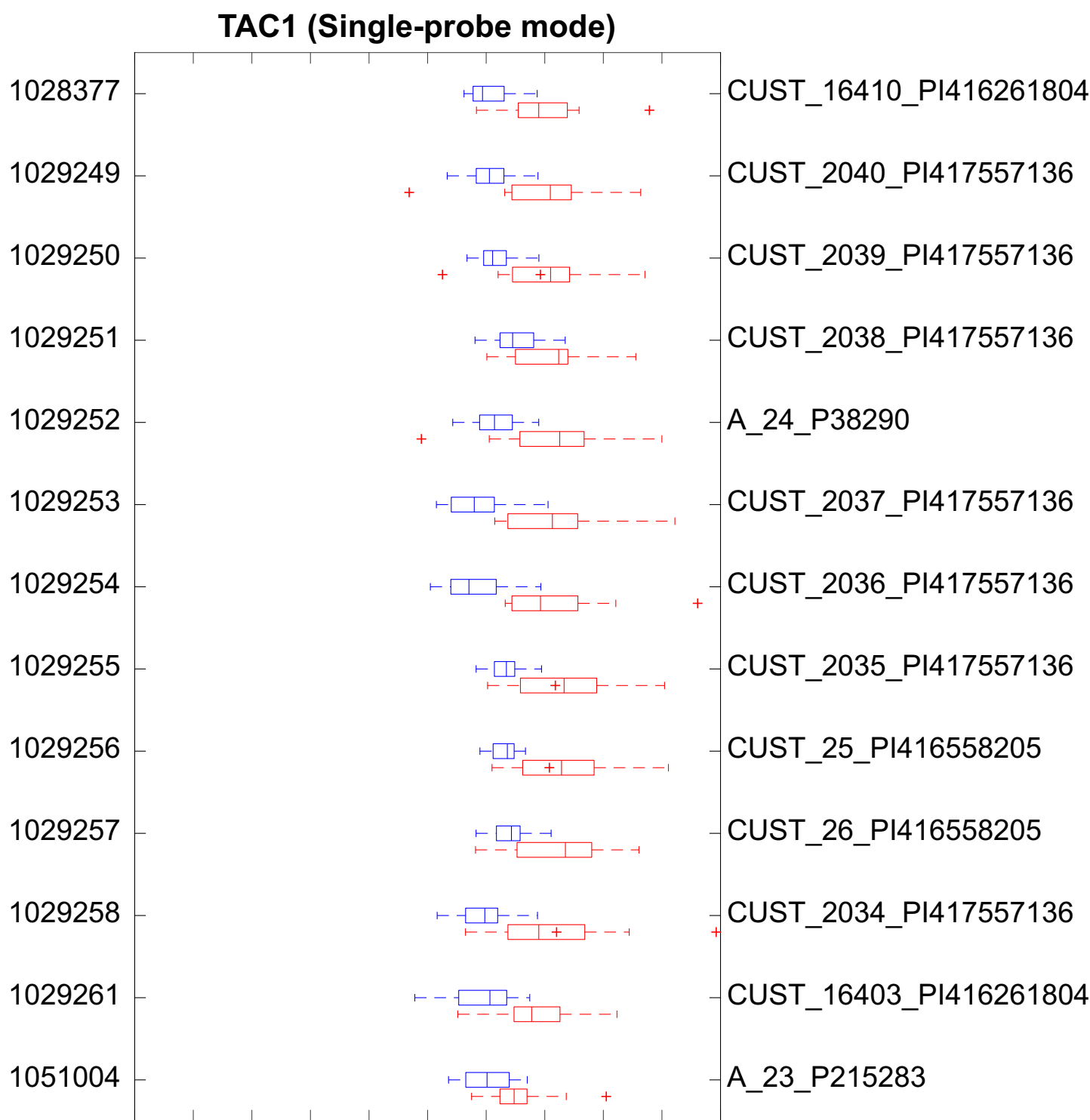

c)

MAOA (Single-probe mode)

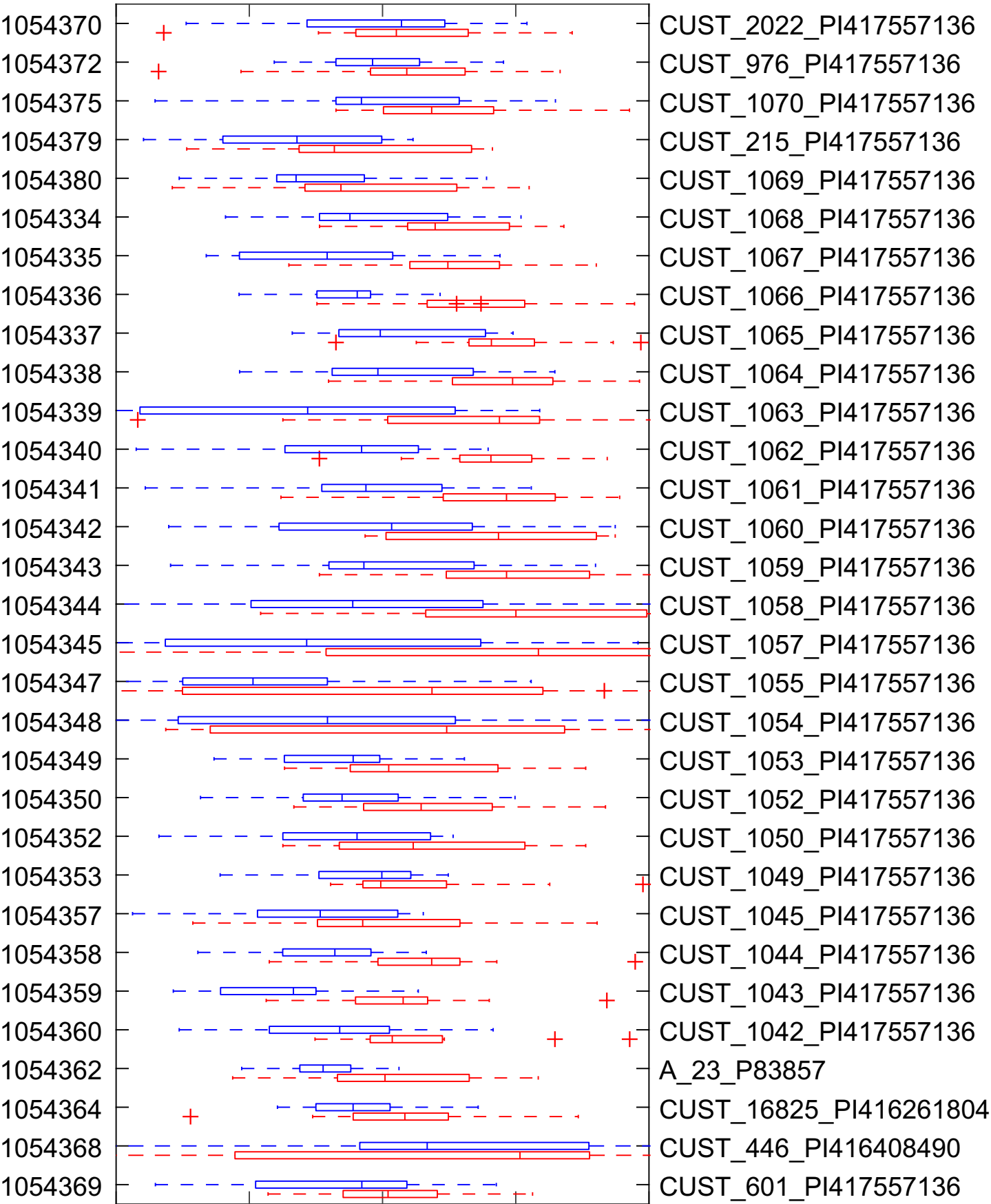

Supplement: Supplementary file 2 — Supplementary Figure 2. Box- and whisker plots of differential gene expressions. (a) Expression levels (z-scores) from the all-probes mode for the 25 investigated candidate genes. TAC1 and MAOA achieved the significant results. Red boxes: Fp2; blue boxes: Fp1; left y-axis: AllenBrain identifier; right y-axis: Entrez gene symbol. (b) Expression levels (z-scores) from the single-probe mode for TAC1, and (c) for MAOA (left y-axis: AllenBrain identifier; right y-axis: oligoprobe identifier) (PDF 1378 KB) [file 429_2018_1620_MOESM2_ESM.pdf]
