# Supplementary material for: Integration of transcriptomic and cytoarchitectonic data implicates a role for MAOA and TAC1 in the limbic-cortical network
Source: Brain Struct Funct. 2018 Feb 24;223(5):2335–42. doi: 10.1007/s00429-018-1620-6 (PMC5968065; doi:10.1007/s00429-018-1620-6)
Supplement: Supplementary file 4 — Supplementary Table 1. Overview of all genes analyzed in the present study. (a) The 25 candidate genes for MDD including oligoprobe identifier (probe_id), gene (gene_symbol), and Entrez gene identifier (entrez_id), (b) the 25 random genes, (c) the 14 color genes and the original references from which they were selected (PDF 208 KB) [file 429_2018_1620_MOESM4_ESM.pdf]

a)

The 25 MDD genes

probe_id,gene_symbol,entrez_id

1059351,"ADRA2A",150

1059352,"ADRA2A",150

1059353,"ADRA2A",150

1058915,"AVPR1B",553

1058914,"AVPR1B",553

1058916,"AVPR1B",553

1029156,"CHRM2",1129

1029155,"CHRM2",1129

1029150,"CHRM2",1129

1029149,"CHRM2",1129

1029146,"CHRM2",1129

1029142,"CHRM2",1129

1029138,"CHRM2",1129

1029136,"CHRM2",1129

1029135,"CHRM2",1129

1029134,"CHRM2",1129

1029133,"CHRM2",1129

1029126,"CHRM2",1129

1029125,"CHRM2",1129

1029124,"CHRM2",1129

1029123,"CHRM2",1129

1029122,"CHRM2",1129

1029121,"CHRM2",1129

1025133,"CHRM2",1129

1023492,"CHRM2",1129

1029129,"CHRM2",1129

1029128,"CHRM2",1129

1029127,"CHRM2",1129

1028516,"CNR1",1268

1028515,"CNR1",1268

1028514,"CNR1",1268

1028513,"CNR1",1268

1028512,"CNR1",1268

1028511,"CNR1",1268

1028510,"CNR1",1268

1028509,"CNR1",1268

1028508,"CNR1",1268

1028507,"CNR1",1268

1028506,"CNR1",1268

1028505,"CNR1",1268

1028504,"CNR1",1268

1028503,"CNR1",1268

1028502,"CNR1",1268

1028484,"CNR1",1268

1028483,"CNR1",1268

1028478,"CNR1",1268

1028477,"CNR1",1268

1028474,"CNR1",1268

1028473,"CNR1",1268

1028472,"CNR1",1268

1028471,"CNR1",1268

1028470,"CNR1",1268

1028469,"CNR1",1268

1028466,"CNR1",1268

1028465,"CNR1",1268

1028464,"CNR1",1268

1028462,"CNR1",1268

1028461,"CNR1",1268

1028458,"CNR1",1268

1028457,"CNR1",1268

1028456,"CNR1",1268

1028455,"CNR1",1268

1028454,"CNR1",1268

1028453,"CNR1",1268

1028452,"CNR1",1268

1028451,"CNR1",1268

1028450,"CNR1",1268

1028448,"CNR1",1268

1028447,"CNR1",1268

1028446,"CNR1",1268

1028445,"CNR1",1268

1028444,"CNR1",1268

1028442,"CNR1",1268

1028441,"CNR1",1268

1028440,"CNR1",1268

1028439,"CNR1",1268

1028438,"CNR1",1268

1028437,"CNR1",1268

1028434,"CNR1",1268

1028433,"CNR1",1268

1028432,"CNR1",1268

1028431,"CNR1",1268

1028430,"CNR1",1268

1028429,"CNR1",1268

1028428,"CNR1",1268

1028427,"CNR1",1268

1028426,"CNR1",1268

1028425,"CNR1",1268

1028424,"CNR1",1268

1015329,"CNR1",1268

1028436,"CNR1",1268

1028435,"CNR1",1268

1028501,"CNR1",1268

1028500,"CNR1",1268

1028499,"CNR1",1268

1028498,"CNR1",1268

1028497,"CNR1",1268

1028494,"CNR1",1268

1028493,"CNR1",1268

1028492,"CNR1",1268

1028491,"CNR1",1268

1028490,"CNR1",1268

1028489,"CNR1",1268

1028488,"CNR1",1268

1028487,"CNR1",1268

1028486,"CNR1",1268

1028485,"CNR1",1268

1028548,"CNR1",1268

1028547,"CNR1",1268

1028542,"CNR1",1268

1028536,"CNR1",1268

1028535,"CNR1",1268

1028534,"CNR1",1268

1028527,"CNR1",1268

1028526,"CNR1",1268

1028518,"CNR1",1268

1028517,"CNR1",1268

1057976,"CREB1",1385

1057975,"CREB1",1385

1057974,"CREB1",1385

1059659,"CREB1",1385

1057967,"CRH",1392

1057966,"CRH",1392

1057965,"CRH",1392

1019360,"CRHR1",1394

1019310,"CRHR1",1394

1013314,"CRHR1",1394

1011485,"CRHR1",1394

1057962,"CRHR2",1395

1057961,"CRHR2",1395

1057960,"CRHR2",1395

1028346,"CUX2",23316

1028345,"CUX2",23316

1028344,"CUX2",23316

1056548,"GAD2",2572

1056547,"GAD2",2572

1056546,"GAD2",2572

1056545,"GAD2",2572

1056544,"GAD2",2572

1013593,"GAD2",2572

1013489,"GAD2",2572

1013359,"GAD2",2572

1013692,"GAD2",2572

1012504,"GAD2",2572

1012098,"GAD2",2572

1011320,"GAD2",2572

1011137,"GAD2",2572

1010770,"GAD2",2572

1010725,"GAD2",2572

1010443,"GAD2",2572

1010639,"GAD2",2572

1056550,"GAD2",2572

1048801,"GPR50",9248

1048800,"GPR50",9248

1024804,"HTR1A",3350

1024803,"HTR1A",3350

1024812,"HTR1A",3350

1055392,"HTR1B",3351

1055381,"HTR1B",3351

1055378,"HTR1B",3351

1055374,"HTR1D",3352

1055373,"HTR1D",3352

1055372,"HTR1D",3352

1055361,"HTR1D",3352

1055357,"HTR1D",3352

1055356,"HTR1D",3352

1055355,"HTR1D",3352

1055354,"HTR1D",3352

1055347,"HTR1D",3352

1055346,"HTR1D",3352

1055345,"HTR1D",3352

1055342,"HTR1D",3352

1055377,"HTR1D",3352

1024617,"HTR3A",3359

1024606,"HTR3A",3359

1024594,"HTR3A",3359

1024593,"HTR3A",3359

1024592,"HTR3A",3359

1021988,"HTR5A",3361

1021983,"HTR5A",3361

1021982,"HTR5A",3361

1021981,"HTR5A",3361

1021979,"HTR5A",3361

1021975,"HTR5A",3361

1021974,"HTR5A",3361

1021973,"HTR5A",3361

1021972,"HTR5A",3361

1021970,"HTR5A",3361

1021969,"HTR5A",3361

1021968,"HTR5A",3361

1054369,"MAOA",4128

1054368,"MAOA",4128

1054364,"MAOA",4128

1054362,"MAOA",4128

1054360,"MAOA",4128

1054359,"MAOA",4128

1054358,"MAOA",4128

1054357,"MAOA",4128

1054353,"MAOA",4128

1054352,"MAOA",4128

1054350,"MAOA",4128

1054349,"MAOA",4128

1054348,"MAOA",4128

1054347,"MAOA",4128

1054345,"MAOA",4128

1054344,"MAOA",4128

1054343,"MAOA",4128

1054342,"MAOA",4128

1054341,"MAOA",4128

1054340,"MAOA",4128

1054339,"MAOA",4128

1054338,"MAOA",4128

1054337,"MAOA",4128

1054336,"MAOA",4128

1054335,"MAOA",4128

1054334,"MAOA",4128

1054380,"MAOA",4128

1054379,"MAOA",4128

1054375,"MAOA",4128

1054372,"MAOA",4128

1054370,"MAOA",4128

1053289,"PDE1A",5136

1053288,"PDE1A",5136

1053287,"PDE1A",5136

1063261,"PDE1A",5136

1023147,"SLC6A2",6530

1023146,"SLC6A2",6530

1051465,"SLC6A4",6532

1051464,"SLC6A4",6532

1051461,"SLC6A4",6532

1051460,"SLC6A4",6532

1051459,"SLC6A4",6532

1051458,"SLC6A4",6532

1051457,"SLC6A4",6532

1051489,"SLC6A4",6532

1051483,"SLC6A4",6532

1051472,"SLC6A4",6532

1051466,"SLC6A4",6532

1025754,"SST",6750

1025749,"SST",6750

1025748,"SST",6750

1025747,"SST",6750

1051004,"TAC1",6863

1029261,"TAC1",6863

1029258,"TAC1",6863

1029257,"TAC1",6863

1029256,"TAC1",6863

1029255,"TAC1",6863

1029254,"TAC1",6863

1029253,"TAC1",6863

1029252,"TAC1",6863

1029251,"TAC1",6863

1029250,"TAC1",6863

1029249,"TAC1",6863

1028377,"TAC1",6863

1050663,"TPH1",7166

1050662,"TPH1",7166

1050661,"TPH1",7166

1035832,"TPH2",121278

1035831,"TPH2",121278

1028612,"HTR2A",3356

1028611,"HTR2A",3356

1028610,"HTR2A",3356

1028609,"HTR2A",3356

1028608,"HTR2A",3356

1028607,"HTR2A",3356

1028606,"HTR2A",3356

1028605,"HTR2A",3356

1028604,"HTR2A",3356

1028603,"HTR2A",3356

1028602,"HTR2A",3356

1028601,"HTR2A",3356

1028634,"HTR2A",3356

1028633,"HTR2A",3356

1028631,"HTR2A",3356

1028627,"HTR2A",3356

1028626,"HTR2A",3356

1028625,"HTR2A",3356

1028620,"HTR2A",3356

1028619,"HTR2A",3356

1028618,"HTR2A",3356

1028617,"HTR2A",3356

1028615,"HTR2A",3356

1028614,"HTR2A",3356

1028613,"HTR2A",3356

b)

The 25 random genes

probe_id,gene_symbol,entrez_id

1039532,"C8orf33",65265

1039531,"C8orf33",65265

1039530,"C8orf33",65265

1046477,"FZD10",11211

1046476,"FZD10",11211

1047556,"TNIP1",10318

1047555,"TNIP1",10318

1020557,"C9orf84",158401

1015178,"C9orf84",158401

1015147,"C9orf84",158401

1021638,"61E3.4",440345

1014742,"61E3.4",440345

1011169,"61E3.4",440345

1011981,"61E3.4",440345

1069865,"61E3.4",440345

1019428,"ASAH1",427

1032727,"ASPM",259266

1032726,"ASPM",259266

1032725,"ASPM",259266

1045100,"PTCD1",26024

1045099,"PTCD1",26024

1045844,"FBXW11",23291

1045843,"FBXW11",23291

1026527,"CBFB",865

1026526,"CBFB",865

1044801,"GREM1",26585

1044800,"GREM1",26585

1019663,"CERKL",375298

1013827,"CERKL",375298

1013746,"CERKL",375298

1041456,"LENEP",55891

1041455,"LENEP",55891

1033718,"HTR3C",170572

1033713,"HTR3C",170572

1033704,"HTR3C",170572

1033702,"HTR3C",170572

1033701,"HTR3C",170572

1033700,"HTR3C",170572

1056207,"GPS2",2874

1056206,"GPS2",2874

1032891,"LCE5A",254910

1032890,"LCE5A",254910

1014716,"AC002472.9",439931

1014210,"AC002472.9",439931

1012353,"AC002472.9",439931

1032756,"KLHL34",257240

1032755,"KLHL34",257240

1057292,"ELAVL2",1993

1057291,"ELAVL2",1993

1015679,"SSR4P1",728039

1013573,"SSR4P1",728039

1031320,"OR5D13",390142

1031319,"OR5D13",390142

1042732,"FNDC8",54752

1042731,"FNDC8",54752

1042092,"TMEM206",55248

1042091,"TMEM206",55248

1056459,"GDF2",2658

1056458,"GDF2",2658

1047601,"SIGMAR1",10280

1047600,"SIGMAR1",10280

c)

The 14 color genes

probe_id,gene_symbol,entrez_id

1059034,"ASIP",434

1059033,"ASIP",434

1042687,"BNC2",54796

1042685,"BNC2",54796

1042684,"BNC2",54796

1042686,"BNC2",54796

1049193,"EIF2S2",8894

1049192,"EIF2S2",8894

1049191,"EIF2S2",8894

1049190,"EIF2S2",8894

1055897,"GSS",2937

1055896,"GSS",2937

1049158,"HERC2",8924

1054961,"IRF4",3662

1054960,"IRF4",3662

1054275,"MC1R",4157

1054274,"MC1R",4157

1054273,"MC1R",4157

1053495,"OCA2",4948

1053494,"OCA2",4948

1024061,"RALY",22913

1024060,"RALY",22913

1024059,"RALY",22913

1027233,"SLC24A4",123041

1027232,"SLC24A4",123041

1032553,"SLC24A5",283652

1032552,"SLC24A5",283652

1043703,"SLC45A2",51151

1043702,"SLC45A2",51151

1050524,"TYR",7299

1050523,"TYR",7299

1050522,"TYR",7299

1019628,"VASH2",79805

1015046,"VASH2",79805

1014927,"VASH2",79805

Year	Journal	Trait	PMID	Implicated genes
2015	Journal of Investigative Dermatology	skin color	25705849	IRF4, MC1R, ASIP, RALY, BNC2
2015	Human Genetics	skin color	25963972	ASIP, SLC45A2, IRF4, HERC2, OCA2, MC1R, EIF2S2, GSS
2016	Nature Communications	hair color	26926045	SLC45A2, IRF4, TYR, HERC2, SLC24A5
2013	Human Molecular Genetics	eye color	23548203	SLC24A4, HERC2, VASH2
2010	PLOS Genetics	hair color	20585627	OCA2, IRF4, SLC45A2, SLC24A4, MC1R
2010	PLOS Genetics	red hair	20585627	MC1R, ASIP
2010	PLOS Genetics	eye color	20585627	OCA2, SLC24A4, IRF4, SLC45A2, TYR
2010	PLOS Genetics	green eyes	20585627	OCA2, SLC24A4, TYR
