# Supplementary material for: Integration of transcriptomic and cytoarchitectonic data implicates a role for MAOA and TAC1 in the limbic-cortical network
Source: Brain Struct Funct. 2018 Feb 24;223(5):2335–42. doi: 10.1007/s00429-018-1620-6 (PMC5968065; doi:10.1007/s00429-018-1620-6)

**Default settings:**

| **a) *Configuration* GUI** | |
| --- | --- |
| Search regions | VOIs (analyzed format, map identifiers according to JuBrain definitions) |
| Donors | Select AllenBrain Donors (Strg+Mouse for multiple selections) |
| Gene list | Select predefined gene list (example: Supplementary Table 1, gene symbols according to Entrez definitions) |
| Output base folder | Parent folder for all subsequent outputs |
| Project name | Freely selectable project name |
| Threshold | threshold value for limiting the VOIs (range: 1-10, default: 2) |
| **b) *Analysis* GUI** | |
| Path definitions Input file | Project file defined in *Configuration* GUI |
| Gene list | Select predefined gene list (example: Supplementary Table 1) |
| Analyze mode | Choose between gene-wide approach (winsorized mean of all available oligoprobes per gene) and transcript-specific approach (individual oligoprobe per transcript) |
| Repetitions | Number of repetitions (permutations) for the gene-level analysis (default: 10,000 rounds) |
| **c) *Visualization*GUI** | |
| Select reference brain | Reference brain for visualization of the tissue blocks |
| Cut of selection | Define parts of the brain which should be cut out for better visualization of the results (default: margin 25) |

a)


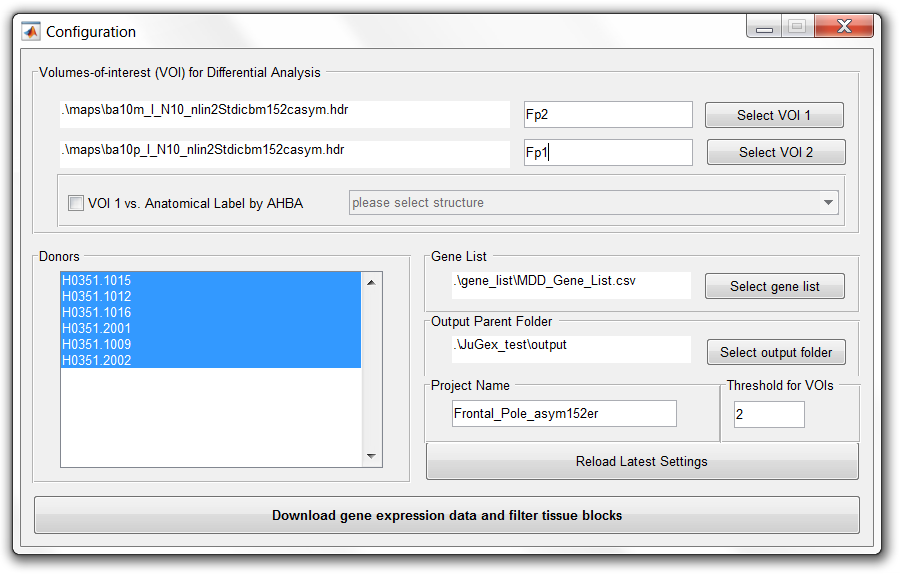


b)


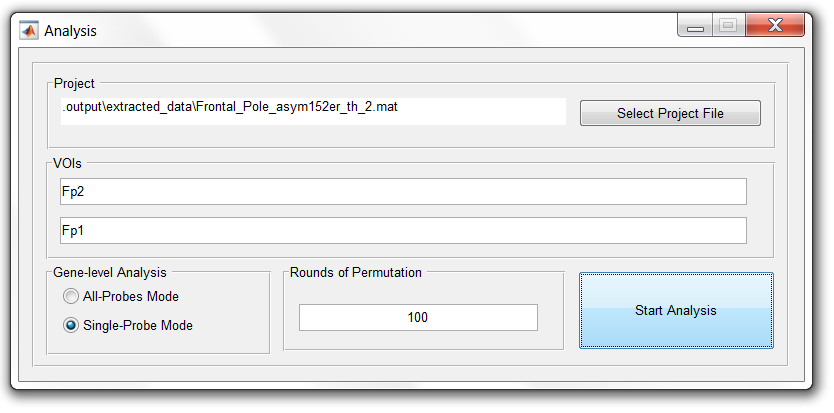


c)


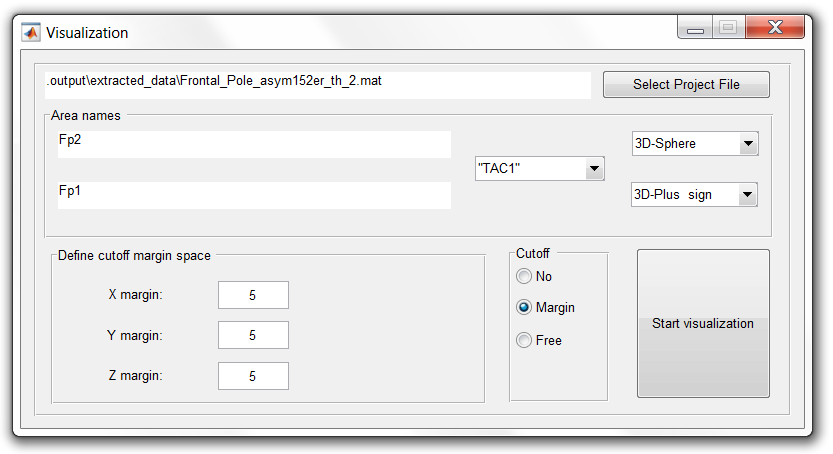

Supplement: Supplementary file 5 — Supplementary Table 2. Description of the default settings used in the standard workflow. (a) GUI Configuration, (b) GUI Analysis, and (c) GUI Visualization (DOCX 136 KB) [file 429_2018_1620_MOESM5_ESM.docx]
