# Supplementary material for: Integration of transcriptomic and cytoarchitectonic data implicates a role for MAOA and TAC1 in the limbic-cortical network
Source: Brain Struct Funct. 2018 Feb 24;223(5):2335–42. doi: 10.1007/s00429-018-1620-6 (PMC5968065; doi:10.1007/s00429-018-1620-6)
Supplement: Supplementary file 6 — Supplementary Table 3. Textual output of the result from the example of use generated by the Matlab console. The index indicates the line number from the input gene list (PDF 17 KB) [file 429_2018_1620_MOESM6_ESM.pdf]

Textual output Matlab console
>>>

```
#########################################
####          Gene-level Analysis           #####
####                                        #####
####          Single-probe mode             #####
#########################################
```

-- n-way ANOVA with user-specified permutations (n= 10,000)

Index	Probe_name	Entrez_ID	Gene_Symbol	p_FWE_corrected
249	CUST_2036_PI417557136	686	'"TAC1"'	0.0475

Number of tissue blocks in Fp2 : 12
Number of tissue blocks in Fp1 : 18
Sum of tissue blocks : 30
Sum of oligoprobes : 285
Number of permutations : 10,000

Elapsed time is 6561.626481 seconds.
